# Consistency of Outputs of the Selected Motion Acquisition Methods for Human Activity Recognition

**DOI:** 10.1155/2019/9873430

**Published:** 2019-07-07

**Authors:** Magdalena Smoleń

**Affiliations:** AGH University of Science and Technology, Kraków 30-059, Poland

## Abstract

The aim of this paper is to choose the optimal motion sensor for the selected human activity recognition. In the described studies, different human motion measurement methods are used simultaneously such as optoelectronics, video, electromyographic, accelerometric, and pressure sensors. Several analyses of activity recognition were performed: recognition correctness for all activities together, matrices of the recognition errors of the individual activities for all volunteers for the individual sensors, and recognition correctness of all activities for each volunteer and each sensor. The experiments enabled to find a range of interchangeability and to choose the most appropriate sensor for recognition of the selected motion.

## 1. Introduction

Telemetric recording and automatic interpretation of motion activities play a significant role in home monitoring. From a variety of applications, we can distinguish a few most common ones: prevention and detection of falls, detection of abnormal or dangerous situations, rehabilitation monitoring, and activity assessment and quantification. An automatic system usually consists of sensors, specific signal or image processing methods, and recognition module for the selected activity. Selection of sensors seems to be the most important issue and must take into account useable sensor properties: wearing ability, sensitivity to disturbances, occurrence of outsiders, etc. Out of the many propositions of sensors, it is difficult to choose the best universal one because each sensor works best in a certain range of recognized activities. This fact motivates us to study that topic.

In [1], electromyographic (EMG) analysis of four lower limb muscles was performed during seven classes of preventive exercises against loss of balance or falling. Other researchers integrated EMG and inertial measurement unit (IMU) to construct a balance evaluation system for recording the body in a dynamic and static posture [2]. In [3], seven hand movements were classified (by neural networks with backpropagation and Gustafson–Kessel algorithm) on the basis of EMG signal of four forearm muscles. An EMG- and augmented reality- (AR-) based rehabilitation system for the upper limbs was proposed in [4]. In [5], an EMG biofeedback device for forearm physiotherapy was constructed to discriminate 6 classes of movements.

Novak et al. [6] proposed a system for automatic detection of gait phases using acceleration and pressure sensors and supervised learning algorithm. For gait abnormalities detection in [7], the authors built a prototype of pressure force sensing resistor (FSR), bend sensor, and IMU. Principal component analysis (PCA) was used for the features generation and support vector machine (SVM) for multiclass classification. Shu et al. [8] presented a time-space measurement tool in the form of insoles of conductive fabric sensors placed around the midfoot and the heel. The wireless capacitive pressure sensors were introduced in [9]. Other studies [10] were related to equilibrium measurements with an instrumented insole with 3 pressure sensors per foot.

An accelerometric (ACC) system for monitoring the daily motor activity (sitting, standing, lying, and periods of natural walking) was proposed in [11]. An ACC sensor was placed on the subject's sternum. Detection of gait parameters by means of a detector composed of gyrometric, accelerometric, and magnetic sensors was proposed in [12]. Rong et al. [13] presented the use of 3D accelerometric sensor located at the waist to identify people based on their characteristic gait patterns. Identification was prepared with discrete wavelet transform (DWT). Jafari et al. [14] proposed ACC-based detection of accidental fall. The selected signal features were used for distinction of four transitions (sitting-standing, standing-sitting, lying-standing, and standing-lying) with the use of neural network and *k*-nearest neighbour (*k*-NN) classification. In [15], researchers developed ACC-based fall detection for smartphones. The proposed system enabled fall event detection, location tracking of the person, and notifications of emergency situations.

Juang et al. [16] introduced a system for detection of four body postures (standing, bending forward, sitting, or lying) and sudden falls. For classification purposes, the silhouette was segmented from each image frame. The feature vector was composed of Fourier transform coefficients and a ratio of body silhouette length and width. Real-time system was implemented in [17]. It consisted of three main modules: segmentation of silhouette, recognition, and identification of posture. The authors introduced decision rules based on body parameters. It was possible to detect four postures: standing, sitting, squatting, and bending. In [18], authors performed analysis by means of supervised and nonsupervised learning for classification of the body position on images sequence. Other researchers [19] presented the posture detection method which took into account information about the body shape and the skin colour. Song and Chen [20] proposed vision-based activity recognition on the basis of information of pose, location, and elapsed time.

In the mentioned papers, the selection of particular sensors was not so clearly justified. This raises a natural question about the optimal choice. The aim of our research was based on the use of various sensors applied to simultaneously capture the signs in basic activities and study the correlation of information obtained from them. This approach enabled the choice of the proper sensor depending on the situation and the current need. The experiments aimed at determining how well the simple measuring devices can approximate the information obtained from the specialized medical equipment. Our measurements were performed by means of three-dimensional motion capture system, wireless EMG amplifier and wireless feet pressure system (as reference equipment), and accelerometer and video camera (as currently available consumer-grade sensors).

## 2. Materials and Methods

### 2.1. Plan of the Experiment

A total number of 20 volunteers (8 women, 12 men, age—22 to 61, average age—27) were examined. Each subject was instructed to do about 30 (19 to 46) repetitions of 12 different activities:

- Squatting from a stand position (1a) and getting up from a squat (1b)
- Sitting on a chair from a stand position (2a) and getting up from a chair (2b)
- Reaching (3a) and returning from reaching the upper limb forward in the sagittal plane (standing) (3b)
- Reaching (4a) and returning from reaching the upper limb upwards in the sagittal plane (standing) (4b)
- Bending from a stand position (5a) and straightening the trunk forward in the sagittal plane (5b)
- Single step for the right (6a) and left lower limb (6b).

The measurements were performed simultaneously with the following:

- A, a motion capture system: Optotrak Certus (NDI) with NDI First Principles software
- B, a wireless biopotential amplifier: ME6000 (Mega Electronics) with MegaWin software
- C, a wireless feet pressure measurement system: ParoLogg with Parologg software
- D, a digital video camera: Sony HDR-FX7E
- E: ACC recorder (Revitus system) with dedicated software.

### 2.2. Characteristics of the Examined Signals

**Figure 1:**
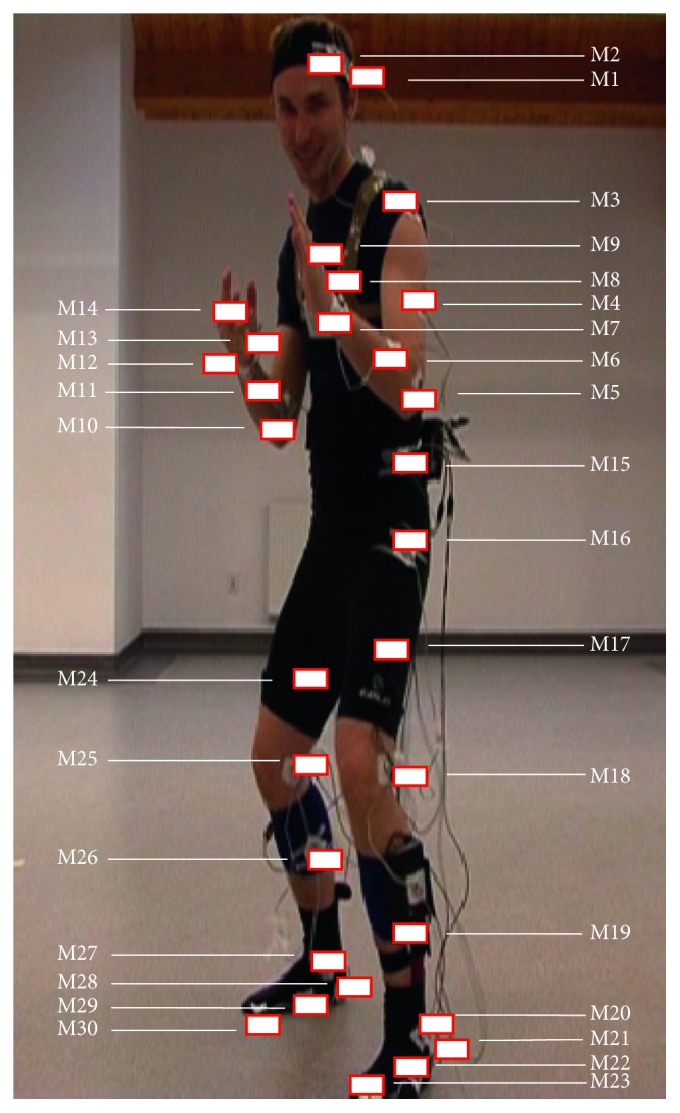
Placement of the markers M1 to M30.

The three-dimensional motion trajectories of 30 infrared markers M1 to M30 located on the body were measured from the left side of the observed person (Figure 1). The acquisition was performed with the sampling frequency 100 Hz, accuracy 0.1 mm, and resolution 0.01 mm.

Surface EMG signals were recorded (2 kHz) from 8 muscles of both lower limbs: (1) quadriceps (vastus lateralis), (2) biceps femoris, (3) tibialis anterior, and (4) gastrocnemius (medial head).

Feet pressure signals were captured with 64 piezoresistive sensors (32 for each feet) with 100 Hz. Triaxial acceleration signal was recorded by sensors integrated in Revitus device located on the sternum. The recorder enabled online measurement via Bluetooth (100 Hz).

Video signals (720 × 576 pixels, 25 frames per second) were obtained from silhouette measurement using a digital camera placed from the volunteer's left side.

### 2.3. Processing of the Measurement Data

To calculate feature vectors for classification, the processing of data recorded with sensors B to E was performed in MATLAB.

The three-dimensional motion trajectories were used for determining the precise time moments of start and end of activities. The exception was the gait (6a, 6b), which cannot be performed in a natural way in the distance as short as 4 m (the maximal width of registration space of the motion capture system). Therefore, for the gait (6a, 6b), the start and end points of duration were determined from visual analysis of video frames.

**Figure 2:**
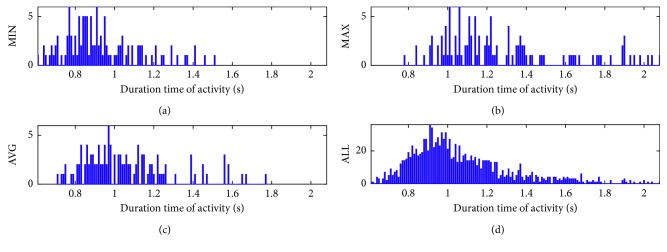
Histograms of performance time for all volunteers and all activities together: (a) minimal MIN, (b) maximal MAX, (c) average AVG duration, and (d) collective ALL.

**Figure 3:**
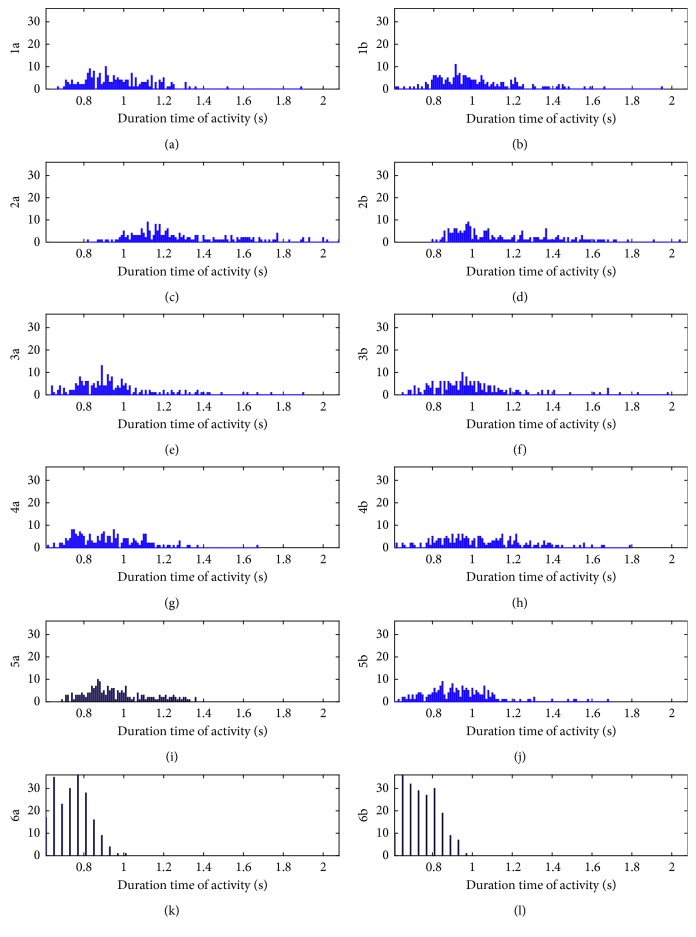
Histograms 1a to 6b of all performance time for all volunteers, for each activity separately 1a to 6b.

The difference of performance time between analyzed movements and acting volunteers requires normalization of the data length with a window *W*. In order to make the optimal selection of its width, a set of histograms of activities performance were calculated:

- Histograms of minimal, maximal, and average (MIN, MAX, AVG) performance time for all people and all activities together; the ALL histogram—for all values of duration time together and for all volunteers and activities (Figure 2)
- Histograms of all performance time for all volunteers, for each activity separately from 1a to 6b (Figure 3).

Based on the ALL histogram, the length of time window was set to *W*=1.6 s, as the shortest of all window-covering activities of various types. Above this value, the other histograms (except for MAX) do not show a significant activity.

The electromyographic signals were processed as follows:

- Calculating the absolute value of the signal
- Averaging the signal in a moving time window (0.1 s)
- Normalizing the amplitude separately for each volunteer—dividing the signal by the maximal value from all measurements of all activities for each volunteer
- Creating the vector data (which are then used as a component of the input classifier vector) consisting of the prepared (as above) EMG signal of each muscle of the left (L) and right (R) lower limb: $\left[\begin{array}{cccccccc} \text{EL}1 & \text{EL}2 & \text{EL}3 & \text{EL}4 & \text{ER}1 & \text{ER}2 & \text{ER}3 & \text{ER}4 \end{array}\right]$
- Normalizing the amplitude to (0 1] interval
- Resampling the signal to the frequency of 25 Hz.

The feet pressure signals were processed as follows:

- Averaging the signal values from the sensors in the three selected areas—the heel (1), the center (2), and the front (3) for the left (*L*) and the right (*R*) foot: $\left[\begin{array}{cccccc} \text{L}1 & \text{L}2 & \text{L}3 & \text{R}1 & \text{R}2 & \text{R}3 \end{array}\right]$
- Averaging the signal in a moving time window of 0.3 s
- Normalizing the amplitude for each volunteer separately
- Creating the vector data: $\left[\begin{array}{cccccc} \text{L}1 & \text{L}2 & \text{L}3 & \text{R}1 & \text{R}2 & \text{R}3 \end{array}\right]$
- Normalizing the amplitude to (0 1] interval
- Resampling the signal to the frequency of 25 Hz.

The accelerometric signals were processed as follows [21]:

- Subtracting the offset value from the signal (offset—average of the 10 s length signal, when a person is in a stationary upright position) separately for each channel (*x*, *y*, *z*) and for each person
- Averaging the signal in a moving time window of 0.2 s
- Normalizing the amplitude for each volunteer separately
- Creating the vector data consisting of a prepared acceleration signal in the axes *x*, *y*, *z*: $\left[\begin{array}{ccc} X & Y & Z \end{array}\right]$
- Normalizing the amplitude to (0 1] interval
- Resampling the signal to the frequency of 25 Hz.

**Figure 4:**
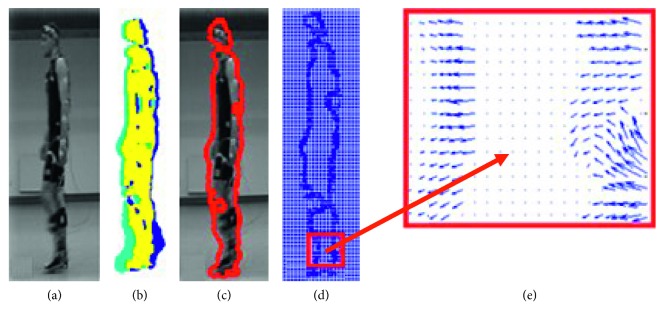
Optical flow algorithm [25]. (a) (*n* − 1)-th frame representing the silhouette during free gait. (b) Joint part (yellow) of the areas: OF_*n*−1/*n*−2_ (blue) and OF_*n*/*n*−1_ (turquoise). (c) (*n* − 1)-th frame representing the free gait with detected silhouette contour. (d) Optical flow calculated on bold contour of the moving silhouette and (e) optical flow of the zoomed silhouette part from red frame in (d).

The video signal was prepared as follows [22]:

- Converting a colour image to a grayscale.
- Calculating the vector motion field with 2 coordinates—optical flow (OF) using Horn–Schunck algorithm [23].
- Median filtering of the motion field components (5 × 5 pixels).
- Detecting the moving objects—binarization of the motion field module with a *T* threshold constant for all people and all activities; the threshold has been chosen experimentally in [24].
- Calculating an area of the moving silhouette *S*_*n*−1_ on the (*n* − 1)-th frame (yellow area in Figure 4(b)) as a joint part from areas OF_*n*−1/*n*−2_ (blue) and OF_*n*/*n*−1_ (turquoise), where OF_*n*−1/*n*−2_ is the motion field calculated on the basis of (*n* − 1)-th and (*n* − 2)-th frame and OF_*n*/*n*−1_is the motion field calculated on the basis of *n*-th and (*n* − 1)-th frame.
- Filling the holes in the area *S*_*n*−1_.
- Thickening the contour mask of the movable silhouette part *S*_*n*−1_ (inside to approximately four pixels (Figure 4(c)).
- Determining the histograms of motion field directions—aggregation of motion field vectors from the bold contour to 8 directions; each direction corresponds to the following angle ranges [−337.50° 22.50°], [22.50° 67.50°],…, [292.50° −337.50°].
- Normalizing the histograms.
- Creating the data vector consisting of the histograms with bins B1, B2, B3, B4, B5, B6, B7, B8—each bar corresponds to one of eight directions: $\left[\begin{array}{cccccccc} \text{B}1 & \text{B}2 & \text{B}3 & \text{B}4 & \text{B}5 & \text{B}6 & \text{B}7 & \text{B}8 \end{array}\right]$.

### 2.4. Identification of the Activities

To identify the selected activities, a supervised classification was performed. The set of all measurement data from each sensor was divided into learning and test sets. The former contained 2400 randomly selected representatives of all 10 activities, while the latter all 4874 remaining cases.

For classification of the selected activities, *k*-NN algorithm and Manhattan metrics were used. Before the classification step, the classifier was tested using the LOO (Leave-One-Out) method. On the basis of these analyses, *k* equal to 1 was the optimal value for all sensors and sets of sensors.

For each activity *a* and each sensor *s*, the correctness of recognition for all volunteers *R*_*s*_*a*_ (1) and its calculation error *U*_*s*_*a*_ (2) were calculated. *U*_*s*_*a*_ is a measure of the results dispersion coming from intersubject differences. Due to different numbers of activity repetitions for each volunteer, we used weighted standard deviation (2):

(1) $$\begin{array}{c} R_{s\text{\_}a}=\frac{P_{s\text{\_}a}}{W_{s\text{\_}a}}, \end{array}$$

where *P*_*s*_*a*_ is the sum of correctly identified repetitions of the activity *a* for all volunteers for the sensor *s* and *W*_*s*_*a*_ is the sum of all repetitions of the activity *a* performed by all volunteers for the sensor *s*:

(2) $$\begin{array}{c} U_{s\text{\_}a}=\sqrt{\frac{\sum_{i=1}^{n}w_{i}{\left(x_{i}-R_{s\text{\_}a}\right)}^{2}}{\left(n-1/n\right)\sum_{i=1}^{n}w_{i}}}, \end{array}$$

where *n* = 20 is the number of weights, equal to the number of volunteers; *w*_*i*_ is the weight for the *i*-th volunteer, equal to the number of the activity *a* repetitions performed by the *i*-th volunteer; and *x*_*i*_ is the percentage of correct recognition for specific activity calculated for the *i*-th volunteer.

In order to represent an additional variable, *R*_*s*_*a*_ALL_ (and its calculation error *U*_*s*_ALL_) was employed. It illustrates the percentage of correct recognition for all activities and all volunteers for each sensor:

(3) $$\begin{array}{c} R_{s\text{\_}a\text{\_ALL}}=\frac{P_{s\text{\_}a\text{\_ALL}}}{W_{s\text{\_}a\text{\_ALL}}}, \end{array}$$

where *P*_*s*_*a*_ALL_ is the sum of correctly identified repetitions of all activities ALL performed by all volunteers for the sensor *s* and *W*_*s*_*a*_ALL_ is the sum of all performed repetitions of all activities ALL for all volunteers.

(4) $$\begin{array}{c} U_{s\text{\_}a\text{\_ALL}}=\sqrt{\frac{\sum_{i=1}^{n}u_{i}{\left(y_{i}-R_{s\text{\_}a\text{\_ALL}}\right)}^{2}}{\left(n-1/n\right)\sum_{i=1}^{n}u_{i}}}, \end{array}$$

where *u*_*i*_ is the weight for the *i*-th volunteer, equal to the total number of repetitions of all activities performed by the *i*-th volunteer, and *y*_*i*_ is the percentage of correct recognition for all activities calculated for volunteer *i*.

For each volunteer *V* and sensor *s*, the percent recognition for all activities *R*_*s*_*V*_ (5) and its calculation error *U*_*s*_*V*_ (6) were determined. *U*_*s*_*V*_ is a measure of the results value dispersion arising from differences between different activities.

(5) $$\begin{array}{c} R_{s\text{\_}V}=\frac{P_{s\text{\_}V}}{W_{s\text{\_}V}}, \end{array}$$

where *P*_*s*_*V*_ is the sum of correctly identified repetitions of all activities with the sensor *s* performed by the volunteer *V* and *W*_*s*_*V*_ is the sum of repetitions of all activities performed by the volunteer *V*.

(6) $$\begin{array}{c} U_{s\text{\_}V}=\sqrt{\frac{\sum_{j=1}^{m}p_{j}{\left(z_{j}-R_{s\text{\_}V}\right)}^{2}}{\left(m-1/m\right)\sum_{j=1}^{m}p_{j}}}, \end{array}$$

where *m* = 12 is the number of weights, equal to the number of activity types, *p*_*j*_ is the weight for the *j*-th activity, equal to the number of its repetitions performed by the volunteer, and *z*_*j*_ is the percentage of correct recognition for the *j*-th activity for the specific subject.

In addition, the calculation error *U*_*s*_*V*_ALL_, was determined as an activity-related dispersion:

(7) $$\begin{array}{c} U_{s\text{\_}V\text{\_ALL}}=\sqrt{\frac{\sum_{j=1}^{m}q_{j}{\left(r_{j}-R_{s\text{\_}a\text{\_ALL}}\right)}^{2}}{\left(m-1/m\right)\sum_{j=1}^{m}q_{j}}}, \end{array}$$

where *q*_*j*_ is the weight for the *j*-th activity equal to the number of all the repetitions performed by all volunteers and *r*_*j*_ is the percentage of correct recognition for the *j*-th activity calculated for all volunteers.

## 3. Results

The correctness of recognition *R*_*s*_*a*_ (1) of activities 1a to 6b for all persons for sensors B to E is presented in Table 1.

Matrices of the recognition errors (in %) of the individual activities 1a to 6b for all volunteers for the individual sensors B to E are shown in Tables 2–5. The percentage of correct recognition *R*_*s*_*a*_ for the individual activities is therefore placed on a diagonal matrix.

The correctness of recognition *R*_*s*_*V*_ of all activities for volunteers *V*1 to *V*20 and *R*_*s*_*a*_ALL_ for ALL volunteers for sensors B to E is presented in Table 6.

## 4. Discussion

The correctness of recognition *R*_*s*_*a*_ (1) is negatively correlated with the dispersion of the value *U*_*s*_*a*_ (2) (Table 1). Therefore, less reliable recognition of the activity carried out by all volunteers does not mean worse recognition of the activity for each individual volunteer, but rather it is the implication of the individual way of performing the activity by the volunteer.

Some types of activities such as free gait or the return from reaching in the vertical and horizontal plane showed much less reliable recognition than others, regardless of the sensor type. Reliability of gait recognition is low probably due to high diversity in walking rhythm. Reaching is difficult to recognize, as it is characterized by low degree of dynamics of the whole body.

It was found that, among the single sensors, the best classifier for different activities is sensor B, followed successively by sensors D, E, and C.

The correctness of recognition *R*_*s*_*V*_ (5) is negatively correlated with the value of dispersion *U*_*s*_*V*_ (7) (Table 6). It means that less reliable recognition for a single volunteer (taking into account all activities) does not come from an inferior recognition reliability of every single activity for that volunteer, but rather it is a result of the existing inconsistency of individual activities recognitions.

Our research is focused on the recognition of only 12 types of daily life activities. The motivation of that choice is mainly based on the following aspects:

- Since the chosen activities are done quite often and are easy to repeat, we limit as much as possible the errors coming from different volunteer performance of the activity and thus the comparison of the sensors is more reliable
- It can be presumed that any activity (even more complex) can be presented by means of the simple (elementary) poses [26].

Although the choice of a proper sensor is a very complex issue, in our studies, we simplify it only to the comparison of motion items. Nevertheless, the final choice of the sensors is precisely related with the application. The following requirements should then be taken into consideration:

- Individual characteristics of the sensor signal
- Size of the registration space
- Sensor accuracy
- Sensor portability and unobtrusiveness
- Cost of the sensor device and reliable software
- Privacy of the supervised person.

The reason for the performance differences for each activity and for each sensor has the source in differences in:

- Speed, range, and way of doing the particular motion
- Anatomy and biomechanics of the volunteer body (physical fitness, strength, endurance, flexibility, way of loading the body weight, etc.).

The above factors have an impact on all of the sensors (B to E).

## 5. Conclusions

The paper presents results of recognition of 12 motor activities in human based on individual interpretation of simultaneous recordings from various sensors. The main finding is that some sensors are more appropriate to the selected activities, while the other sensors show higher performance compared with the others. Consequently, we specified both areas where sensors show distinctive properties and a common range of activities where the sensors show similar metrological properties and may be selected based on other criteria (e.g., cost and commodity).

Additionally, we found that some recognition results generalized for all volunteers as well as those generalized for all activities showed surprisingly low values. This suggests that the recognition performance is dependent on particular volunteer (i.e., subject-specific) and also on particular action. Accordingly, the hierarchy of expected recognition results for particular actions is not universal, and to produce optimal results, it should be individually adjusted with regard to particular user behavior.

The prospective ways of future extension of our studies are as follows:

- Expanding the list of activities with more complex ones
- Evaluating and adaptating the proposed solutions in home environment
- Extending video processing algorithm with a detection of individual body parts.

## Figures and Tables

**Table 1:** Correctness of recognition *R*_*s*_*a*_ (in %) of activities 1a to 6b and *R*_*s*_*a*_ALL_ of ALL activities for all volunteers for sensors B to E.

	1a	1b	2a	2b	3a	3b	4a	4b	5a	5b	6a	6b	All
B	96.9	100.0	99.5	98.5	99.0	99.3	99.1	98.6	97.9	98.2	96.0	97.6	98.4
	*3.9*	*0.0*	*1.5*	*3.7*	*3.4*	*1.8*	*3.2*	*3.1*	*3.7*	*3.5*	*12.7*	*7.5*	*1.8*

C	90.8	91.8	95.7	96.7	94.9	92.4	95.3	93.6	87.0	88.2	94.9	97.1	93.1
	*10.0*	*10.8*	*12.4*	*15.5*	*6.9*	*6.4*	*10.2*	*7.6*	*15.0*	*14.6*	*14.1*	*8.2*	*5.7*

D	95.2	97.2	95.5	94.2	96.6	95.1	98.4	97.6	97.9	99.3	96.5	96.0	96.7
	*17.2*	*11.2*	*15.8*	*15.4*	*6.6*	*8.8*	*3.2*	*4.1*	*2.8*	*1.9*	*12.3*	*12.1*	*5.2*

E	99.7	99.5	95.5	95.5	99.3	97.6	96.0	79.8	99.3	99.3	91.7	92.3	95.5
	*1.1*	*1.6*	*17.7*	*18.8*	*1.8*	*4.4*	*7.9*	*25.0*	*1.7*	*1.7*	*9.1*	*8.4*	*4.4*

Calculation errors *U*_*s*_*a*_ and *U*_*s*_*a*_ALL_ are in italics.

**Table 2:** Matrix of recognition errors (in %) of activities 1a to 6b for all volunteers for sensor B.

B		Performed activity											
		1a	1b	2a	2b	3a	3b	4a	4b	5a	5b	6a	6b
Recognized activity	1a	**96.9**		0.5	0.8								
	1b		**100**										
	2a	2.0		**99.5**	0.8	0.2							
	2b				**98.5**								
	3a					**99.0**			0.2				
	3b						**99.3**		0.5				
	4a	0.5				0.2		**99.1**	0.7	1.1			
	4b						0.7	0.7	**98.6**	0.9	1.8		
	5a	0.5				0.5		0.2		**97.9**			
	5b										**98.2**		
	6a											**96.0**	2.4
	6b											4.0	**97.6**

**Table 3:** Matrix of recognition errors (in %) of activities 1a to 6b for all volunteers for sensor C.

C		Performed activity											
		1a	1b	2a	2b	3a	3b	4a	4b	5a	5b	6a	6b
Recognized activity	1a	**90.8**	0.3			4.1		0.5		0.5			
	1b		**91.8**				3.9		0.9				
	2a	0.3	0.8	**95.7**	1.5				0.7	0.2	0.7		
	2b	1.8		1.3	**96.7**				0.2	0.9	1.4		
	3a	3.1				**94.9**	0.2			0.5			
	3b		2.0				**92.4**			0.5			
	4a	2.8	0.5	0.8	1.0	1.0	0.2	**95.3**	4.0	4.6	1.6		
	4b	0.3	3.3	1.5	0.5		1.7	3.8	**93.6**	1.6	5.5		
	5a	1.0		0.3				0.5		**87.0**	2.7	0.3	0.3
	5b		1.3	0.5	0.3		1.5		0.5	4.3	**88.2**		
	6a											**94.9**	2.7
	6b											4.8	**97.1**

**Table 4:** Matrix of recognition errors (in %) of activities 1a to 6b for all volunteers for sensor D.

D		Performed activity											
		1a	1b	2a	2b	3a	3b	4a	4b	5a	5b	6a	6b
Recognized activity	1a	**95.2**	1.0	0.3						0.9			
	1b	2.0	**97.2**	0.3	0.8						0.7		
	2a	0.5	0.5	**95.5**	3.5			0.2	0.2	0.9			0.3
	2b	1.0		2.3	**94.2**					0.2			
	3a					**96.6**		0.7					
	3b						**95.1**		1.2				
	4a			0.3	0.3	2.9	0.2	**98.4**	0.9			0.5	0.5
	4b		0.3	0.3	0.3	0.5	4.6	0.7	**97.6**				
	5a	1.3	0.3	1.0	1.0					**97.9**			
	5b		0.8	0.3							**99.3**		
	6a											**96.5**	3.2
	6b											2.9	**96.0**

**Table 5:** Matrix of recognition errors (in %) of activities 1a to 6b for all volunteers for sensor E.

E		Performed activity											
		1a	1b	2a	2b	3a	3b	4a	4b	5a	5b	6a	6b
Recognized activity	1a	**99.7**								0.5			
	1b		**99.5**								0.7		
	2a			**95.5**			0.2	0.2	0.2				
	2b				**95.5**	0.2	0.2		0.9				
	3a				0.5	**99.3**		3.8	0				
	3b			0.8			**97.6**		2.8				
	4a			3.8	3.8	0.5	0.2	**96.0**	16.2	0.2			
	4b	0.3					1.7		**79.8**				
	5a				0.3					**99.3**			
	5b		0.5								**99.3**		
	6a											**91.7**	7.7
	6b											8.3	**92.3**

**Table 6:** Correctness of recognition *R*_*s*_*V*_ (in %) of all activities for volunteers *V*1 to *V*20 and *R*_*s*_*a*_ALL_ for ALL volunteers for sensors B to E.

	*V*1	*V*2	*V*3	*V*4	*V*5	*V*6	*V*7	*V*8	*V*9	*V*10	*V*11	*V*12	*V*13	*V*14	*V*15	*V*16	*V*17	*V*18	*V*19	*V*20	All
B	99.1	94.8	98.4	98.3	98.7	99.1	99.6	97.7	98.3	98.0	98.4	99.6	92.6	100.0	99.6	99.1	97.3	99.6	99.6	100.0	98.4
	*2.0*	*7.8*	*2.4*	*4.6*	*3.1*	*1.9*	*1.5*	*4.1*	*3.3*	*4.5*	*3.7*	*1.4*	*15.8*	*0.0*	*1.4*	*2.9*	*4.3*	*1.1*	*1.1*	*0.0*	*1.1*

C	98.2	94.8	98.0	97.9	92.3	97.4	97.3	94.9	95.7	91.2	94.4	88.0	85.3	97.1	87.3	88.9	75.2	94.7	93.5	98.4	93.1
	*2.5*	*5.6*	*3.3*	*2.2*	*11.6*	*5.9*	*5.0*	*7.8*	*4.1*	*12.5*	*7.2*	*17.2*	*15.9*	*3.3*	*13.4*	*13.6*	*22.7*	*13.1*	*11.8*	*4.4*	*3.3*

D	96.9	93.1	99.2	96.9	98.3	98.7	100.0	76.2	93.1	97.6	99.2	99.2	91.1	98.2	100.0	98.3	94.6	100.0	100.0	100.0	96.7
	*3.9*	*11.1*	*1.9*	*6.5*	*2.5*	*3.2*	*0.0*	*33.8*	*11.3*	*4.1*	*2.7*	*1.9*	*17.2*	*2.9*	*0.0*	*2.5*	*9.1*	*0.0*	*0.0*	*0.0*	*1.5*

E	97.4	95.3	95.9	88.8	97.9	99.1	99.6	97.7	94.4	93.6	99.2	81.0	96.5	94.9	99.2	98.3	92.3	97.3	97.7	94.8	95.5
	*6.4*	*9.7*	*7.4*	*23.4*	*3.6*	*1.9*	*2.0*	*3.1*	*10.5*	*14.7*	*1.9*	*31.8*	*5.3*	*16.2*	*1.9*	*3.9*	*16.1*	*4.7*	*4.5*	*11.2*	*5.8*

Calculation errors *U*_*s*_*V*_ and *U*_*s*_*V*_ALL_ are in italics.

## Data Availability

Research data are not openly available because of the volunteers' privacy.
